# Complete Genome Sequence of *Citrobacter* Phage CVT22 Isolated from the Gut of the Formosan Subterranean Termite, *Coptotermes formosanus* Shiraki

**DOI:** 10.1128/genomeA.00408-15

**Published:** 2015-07-16

**Authors:** Chinmay Vijay Tikhe, Thomas M. Martin, Chris R. Gissendanner, Claudia Husseneder

**Affiliations:** a Department of Entomology, Louisiana State University Agricultural Center, Baton Rouge, Louisiana, USA; b Department of Basic Pharmaceutical Sciences, School of Pharmacy, University of Louisiana at Monroe, Monroe, Louisiana, USA

## Abstract

The complete genome of bacteriophage CVT22 infecting *Citrobacter* sp. strain TM1552 is reported here. Both the bacteriophage and *Citrobacter* sp. TM1552 were isolated from the gut of the Formosan subterranean termite, *Coptotermes formosanus*. This is the first report of a genome sequence of a bacteriophage isolated from the termite gut.

## GENOME ANNOUNCEMENT

The vital and multifarious bacterial community of the Formosan subterranean termite gut makes a niche for bacteriophages, which remain unstudied to date. Here, we report the first genome sequence of a termite gut bacteriophage (CVT22). CVT22 infects *Citrobacter* sp. strain TM1552 (GenBank accession no. KP765691), also isolated from the termite gut.

Gut homogenate was filtered through a 0.22-µm syringe filter to isolate bacteriophage CVT22, which infected *Citrobacter* sp. TM1552 with clear plaque morphology. DNA was purified from high-titer lysates of CVT22 using phenol-chloroform-isoamyl alcohol extraction and sequenced using the Illumina MiSeq platform (2 × 300 bp) at Molecular Research LP (Shallowater, TX). Sequencing resulted in 2,012,032 reads, with an average read length of 300 bp and approximately 12,000× genome coverage. The reads were assembled using the DNAStar SeqMan NGen DNA assembler. The assembled contig contained terminally redundant repeats, and the genome was confirmed to be circularly permuted by restriction enzyme analysis. Gene predictions were generated using Glimmer (1, 2) and GeneMark (3) and manually annotated with DNA Master (http://cobamide2.bio.pitt.edu/). Phage morphology was determined using electron microscopy (EM). A phage family search was carried out using VIRFAM (4).

The circularly permuted genome of CVT22 is 47,636 bp, with a G+C content of 41.6%. We organized the CVT22 genome into two convergent transcriptional units. Whole-genome nucleotide BLAST using high-similarity criteria against the GenBank nr nucleotide database did not result in any matches. Less-stringent discontiguous MegaBlast showed a match to *Pseudomonas* phage PA11 (query coverage, 11%; identity, 69%) and *Salinivibrio* phage CW02 (query coverage, 11%; identity, 67%). The genome contains 82 predicted protein-coding genes, with 37 (45.12%) exhibiting similarity to phage genes in the GenBank nr protein database. Out of those, 14 were similar (identity, 33 to 68%) to *Pseudomonas* phage PA11 (5), while 11 showed similarity (25 to 65%) to *Salinivibrio* phage CW02 (6). Twenty-five genes (30.48%) were assigned a putative function based on homology. In addition to structural genes, we identified a terminase gene and a lysis cassette consisting of endolysin, holin, and o- and i-spanin genes. Other putative proteins include DNA polymerase, primase/helicase, ATP grasp protein, sigma transcription factor, amido-ligase, *S*-adenosylmethionine-dependent methyltransferase superfamily protein, and aspartate aminotransferase superfamily protein. Two copies each of exonuclease-, endonuclease-, and amidotransferase-encoding genes were also identified. BLAST analysis did not identify any synteny to prophage genomes, and we did not identify any genes encoding proteins involved in lysogeny. This, along with the clear plaque morphology, suggests that CVT22 may have a lytic life cycle. VIRFAM predicted CVT22 to be a member of the *Podoviridae* type 3 group and clustered with *Pseudomonas* phage PA11 (4, 5). The overall size and genome organization of CVT22 is similar to that of PA11, and EM analysis supports the assignment of CVT22 to the *Podoviridae* family.

This is the first description of a genome of a bacteriophage isolated from the termite gut. Further studies of CVT22 will reveal its role in the termite gut microbial ecosystem.

### Nucleotide sequence accession number.

The complete annotated sequence of the *Citrobacter* phage CVT22 genome can be accessed under the GenBank accession no. KP774835.
